# Shifting Towards Empagliflozin First‐Line Therapy in Glycogen Storage Disease Type Ib: A Nationwide Real‐World Study

**DOI:** 10.1002/jimd.70198

**Published:** 2026-05-03

**Authors:** Sema Kalkan Uçar, Neslihan Önenli Mungan, Gülden Fatma Gökçay, Ayşe Çiğdem Aktuğlu Zeybek, Sebile Kılavuz, Seda Güneş, Fatih Kardaş, Pelin Teke Kısa, Kısmet Çıkı, Aynur Küçükçongar Yavaş, Ayça Burcu Kahraman, Merve Yoldaş Çelik, Ebru Canda, Deniz Kor, Meryem Karaca, Tanyel Zübarioğlu, Emine Genç, Havva Yazıcı, Fatma Derya Bulut, Şebnem Kılıç, Hanım Aghakıshılı, Fehime Erdem Karapınar, Ezgi Burgaç, Ertuğrul Kıykım, Alperen Elek, Nihal Kardaş, Ekrem Ünal, Tuba Hilkay Karapınar, Meryem Albayrak, Canan Albayrak, Ayşegül Ünüvar, Saskia B. Wortmann, Deniz Yılmaz Karapınar

**Affiliations:** ^1^ Division of Pediatric Nutrition and Metabolism, Department of Pediatrics Ege University Faculty of Medicine İzmir Türkiye; ^2^ Division of Pediatric Nutrition and Metabolism, Department of Pediatrics Cukurova University Faculty of Medicine Adana Türkiye; ^3^ Division of Pediatric Nutrition and Metabolism, Department of Pediatrics İstanbul University Faculty of Medicine İstanbul Türkiye; ^4^ Division of Pediatric Nutrition and Metabolism, Department of Pediatrics İstanbul University Cerrahpaşa Faculty of Medicine İstanbul Türkiye; ^5^ Division of Pediatric Metabolism and Nutrition, Department of Pediatrics Marmara University Faculty of Medicine İstanbul Türkiye; ^6^ Division of Pediatric Nutrition and Metabolism, Department of Pediatrics, Faculty of Medicine Harran University Şanlıurfa Türkiye; ^7^ Division of Pediatric Metabolism and Nutrition, Department of Pediatrics Erciyes University Faculty of Medicine Kayseri Türkiye; ^8^ Division of Pediatric Metabolism and Nutrition, Department of Pediatrics Dokuz Eylul University Faculty of Medicine İzmir Türkiye; ^9^ Pediatric Metabolism Unit, Department of Pediatrics, Faculty of Medicine Hacettepe University Ankara Türkiye; ^10^ Department of Pediatric Metabolic Diseases Ankara Bilkent City Hospital Ankara Türkiye; ^11^ Division of Pediatric Metabolism Konya City Hospital, University of Health Sciences Konya Türkiye; ^12^ Department of Pediatric Metabolism Adana City Training and Research Hospital Adana Türkiye; ^13^ Department of Pediatrics, Division of Pediatric Metabolism and Nutrition Van Research and State Hospital Van Türkiye; ^14^ Ege University Faculty of Medicine İzmir Türkiye; ^15^ Division of Pediatric Hematology, Department of Pediatrics Ege University Faculty of Medicine İzmir Türkiye; ^16^ Faculty of Health Sciences Medical Point Hospital, Hasan Kalyoncu University Gaziantep Türkiye; ^17^ Department of Pediatric Hematology Oncology Dr. Behcet Uz Pediatric Diseases and Surgery Training and Research Hospital İzmir Türkiye; ^18^ Department of Pediatric Hematology Oncology Kırıkkale University Faculty of Medicine Kırıkkale Türkiye; ^19^ Division of Pediatric Hematology and Oncology, Department of Pediatrics, Faculty of Medicine Ondokuz Mayis University Samsun Türkiye; ^20^ Division of Pediatric Hematology and Oncology, Department of Pediatrics, Faculty of Medicine Istanbul University Istanbul Türkiye; ^21^ University Children's Hospital Paracelsus Medical University Salzburg Austria

**Keywords:** empagliflozin, first‐line monotherapy, glycogen storage disease type Ib (GSD Ib)

## Abstract

Neutrophil dysfunction and neutropenia are burdensome findings in glycogen storage disease type Ib (GSDIb). Treatment with granulocyte‐colony stimulating factor (G‐CSF) often corrects neutropenia but fails to improve clinical symptoms like inflammatory bowel disease (IBD). Recently, empagliflozin (EMPA) was shown to correct the neutrophil dysfunction and its clinical consequences. It is increasingly used as first‐line monotherapy, but long‐term, real‐world data are lacking. This nation‐wide retrospective study investigated 42 GSD1b patients (36 children) treated with EMPA as first‐line monotherapy and compared them with those receiving combination therapy with G‐CSF and treatment‐naïve patients (I:EMPA first‐line monotherapy [*n* = 9]; II:G‐CSF monotherapy [*n* = 7]; III:EMPA plus G‐CSF [*n* = 16]; and IV: neither EMPA nor GCSF [*n* = 10]). Pediatric (P) patients were evaluated separately. In pediatric patients receiving EMPA as first‐line monotherapy (P‐I) and those with EMPA plus G‐CSF (P‐III), the frequency of infections, hospital admissions, and IBD was significantly lower than in patients receiving GCSF‐monotherapy (P‐II) or no treatment (P‐IV). Additionally, significantly improved weight gain was observed in P‐I. While clinical improvement related to correction of neutrophil dysfunction was seen with EMPA first‐line monotherapy (P‐I), significant improvement in absolute neutrophil count (ANC) and hemoglobin levels was only seen in P‐III with additional G‐CSF treatment. In three cases, EMPA was safely paused and subsequently resumed, for example, during pregnancy or liver transplantation. First‐line EMPA monotherapy effectively corrects clinical symptoms of neutrophil dysfunction in GSD Ib patients, even in the absence of a statistically significant increase in ANC.

**Abbreviations**
1,5‐AG: 1,5‐anhydroglucitol
1,5AG6P: 1,5‐anhydroglucitol‐6‐phosphate
A: adults
AEs: adverse events
ALP: alkaline phosphatase
ALT: alanine aminotransferase
ANC: absolute neutrophil count
AST: aminotransferase
B: before treatment with empagliflozin/granulocyte‐colony stimulating factor or baseline for naïve patients
D: during treatment with empagliflozin/granulocyte‐colony stimulating factor or follow‐up period for naïve patients
EMPA: empagliflozin
G6PC3: glucose‐6‐phosphatase catalytic subunit 3 deficiency
G6PT: glucose‐6‐phosphate translocase
G‐CSF: granulocyte‐colony stimulating factor
GSD Ib: glycogen storage disease type Ib
IBD: inflammatory bowel disease
Inf: infants
IQR: interquartile range
P: pediatric
PCDAI: Pediatric Crohn's Disease Activity Index
SDS: standard deviation score
SGLT2: sodium–glucose co‐transporter 2
UTIs: urinary tract infections

## 1 Introduction

Glycogen storage disease type Ib (GSD Ib, MIM #232220) is a rare autosomal recessive disorder caused by pathogenic biallelic variants in the *SLC37A4* gene encoding the transmembrane protein glucose‐6‐phosphate translocase (G6PT) [1, 2]. In addition to hypoglycemia and hepatomegaly, patients with GSD Ib also suffer from neutropenia and neutrophil dysfunction, leading to burdensome complications such as recurrent bacterial infections, mucocutaneous lesions, and inflammatory bowel disease (IBD) [3]. Treatment with granulocyte‐colony stimulating factor (G‐CSF) was used to correct neutropenia but regularly failed to improve the clinical symptoms of neutrophil dysfunction [4].

Only recently the accumulation of 1,5‐anhydroglucitol‐6‐phosphate (1,5‐AG6P), a close structural analogue of glucose‐6‐phosphate and an inhibitor of hexokinases, has been shown to underly neutrophil dysfunction in GSD Ib (and glucose‐6‐phosphatase catalytic subunit 3 (G6PC3) deficiency) [5, 6]. Empagliflozin (EMPA), a sodium–glucose cotransporter 2 (SGLT2) inhibitor, can reduce renal reabsorption and thereby blood concentration of 1,5‐AG and was shown to decrease levels of the toxic metabolite 1,5‐AG6P within neutrophils [5, 6, 7].

This enabled the pathophysiology‐based treatment of neutrophil dysfunction and neutropenia in patients with GSD Ib and G6PC3 deficiency, a major breakthrough [7, 8].

Given the rarity of GSD 1b and the novelty of the treatment, mainly case reports, series, and reviews [9, 10, 11, 12, 13, 14, 15] covering the treatment of about 150 patients are available. Studies investigating EMPA monotherapy initiated as a first‐line treatment, in direct comparison with other therapeutic approaches, are particularly lacking, as are studies evaluating the effect of EMPA on absolute neutrophil count (ANC). With a nation‐wide approach, this study addresses these gaps in knowledge.

## 2 Materials and Methods

### 2.1 Study Design, Ethical Consideration, and Patient Recruitment

This was an observational, retrospective study based on a nation‐wide research collaboration initiated in 2020 by the National Society of Pediatric Nutrition and Metabolism, along with the Congenital Neutropenia Working Group of the Pediatric Hematology Association in Türkiye.

The protocol, consent form, and patient information sheet received approval from the Medical Ethics Committee of Ege University (E‐99166796‐050.04‐1717668). The study was conducted in compliance with the Declaration of Helsinki and ICH‐GCP guidelines; written informed consent was obtained from all participants or their legal guardians.

All patients with genetically confirmed GSD Ib consenting for participation were included by their treating physician who completed a case report form. EMPA treatment in patients with GSD Ib in Türkiye is prescribed and supervised by physicians specialized in inherited metabolic disorders, while G‐CSF is prescribed by hematologists. Initiation of EMPA therapy is individualized, taking into account patient age, clinical status, and caregiver preparedness. In general, treatment is initiated in a hospital setting in infants and younger children to allow close monitoring for hypoglycemia and metabolic instability, whereas in older and clinically stable patients, initiation may be performed in the outpatient setting.

All data had been generated during regular follow up at 13 centers (Adana, Ankara, Kayseri, Kırıkkale, Konya, İstanbul, İzmir, Samsun, Sanliurfa, and Van [Figure S1]) across Türkiye. The data were collected for the period 2020–2024 (last data entry 1st of March 2024).

Patients were stratified into four groups according to treatment regimen (I: EMPA as first‐line monotherapy, II: G‐CSF monotherapy, III: G‐CSF plus EMPA, and IV: neither G‐CSF nor EMPA [treatment‐naïve]). The final validated treatment distribution in the whole cohort was as follows: Group I, *n* = 9; Group II, *n* = 7; Group III, *n* = 16; and Group IV, *n* = 10, totaling 42 patients. The cohort was further categorized by age into infants (Inf) (*n* = 6), pediatric (P) patients (*n* = 30), and adults (A) (*n* = 6). Pediatric (P) patients were evaluated separately. Among pediatric patients (*n* = 30), the distribution across treatment groups was as follows: P‐I (EMPA, *n* = 7), P‐II (G‐CSF, *n* = 5), P‐III (EMPA + G‐CSF, *n* = 11), and P‐IV (treatment‐naïve, *n* = 7). Four of the patients included in III have been previously described [16, 17].

### 2.2 Clinical and Laboratory Assessment via Case Record Form

The following data were collected at baseline, early follow‐up (approximately 1–3 months), and during routine clinical visits thereafter (typically every 3–6 months): anthropometric data; findings upon physical examination (e.g., hepatomegaly); symptoms of neutrophil dysfunction such as oral infections, urogenital infections, perianal ulcers, abscesses, soft tissue infections, pneumonia, upper respiratory tract infections (URTI), urinary tract infections (UTI), central nervous system (CNS) infections, and pancreatitis including information if patients required hospital admission for treatment.

For treated patients, baseline (“at beginning”) was defined as the evaluation performed prior to initiation of the respective treatment regimen under study. For treatment‐naïve patients, baseline referred to the first clinical assessment included in the study period.

Infection frequency was calculated as the number of clinically significant infectious episodes recorded per patient during the defined observation period. Severe infections were defined as infections requiring medical intervention and/or hospitalization. Hospital admissions were quantified as the number of admissions per patient during the same observation period. For treated patients, this period corresponded to treatment duration; for untreated patients, it corresponded to the available follow‐up period within the study timeframe.

Data on IBD was collected using the weighted Pediatric Crohn's Disease Activity Index (wPCDAI) [16]. A wPCDAI score greater than 12.5 was considered indicative of active disease, whereas a score of ≤ 12.5 was considered remission. Parameters of metabolic control collected included: blood glucose, lactate, triglycerides; aminotransferase (AST), alanine aminotransferase (ALT), and alkaline phosphatase (ALP). Hematological parameters collected included ANC and hemoglobin levels.

Additionally, all details on diet/nutritional therapy, details on dosage and duration of EMPA, and G‐CSF were collected as well as all concomitant medications and any other therapies (e.g., solid organ transplantation) or conditions (e.g., pregnancy) were also recorded.

### 2.3 Statistical Analysis

Data were analyzed using R software, version 4.4.3 (R Foundation for Statistical Computing, Vienna, Austria). The normality of continuous variables was assessed using the Shapiro–Wilk test. Based on the data distribution, parametric tests were applied to normally distributed variables, whereas non‐parametric tests were used for non‐normally distributed variables. For comparisons between two time points, the paired *t*‐test was used for normally distributed continuous variables, while the Wilcoxon signed‐rank test was applied for non‐normally distributed variables. For comparisons across three time points, repeated‐measures ANOVA was used for normally distributed data, whereas the Friedman test was used for non‐normally distributed data. When appropriate, post hoc pairwise comparisons were performed with Bonferroni correction. Categorical variables were compared using the chi‐square test or Fisher's exact test when expected cell counts were low. Descriptive statistics for continuous variables were presented as mean ± standard deviation (SD) for approximately normally distributed data and as median with interquartile range (IQR) for non‐normally distributed data. Categorical variables were summarized as frequencies and percentages. A *p* value ≤ 0.05 was considered statistically significant.

## 3 Results

A total of 42 patients (18 female) were included in the four subgroups: I: EMPA first‐line monotherapy (*n* = 9); II: G‐CSF monotherapy (*n* = 7); III: EMPA plus G‐CSF (*n* = 16); IV: no treatment (*n* = 10). Median follow‐up duration was comparable across groups, with values of 24 months (3–43) in Group I, 25 months (24–48) in Group II, 26.5 months (9–47) in Group III, and 25.5 months (5–50) in Group IV. The 36 children (age distribution in Table S1) were additionally investigated as one subgroup: P‐I (*n* = 7); P‐II (*n* = 5); P‐III (*n* = 11); P‐IV (*n* = 7). The genotypes can be found in Table S2.

### 3.1 Three Patients With GSD Ib With Specific Conditions

An 11‐year‐old patient had been treated with empagliflozin (EMPA) for approximately 2 years, achieving marked improvement in neutropenia and neutrophil dysfunction, with complete resolution of recurrent oral ulcers and no infection‐related hospitalizations. The patient had a complex clinical history, including recurrent infections, IBD, and long‐term G‐CSF use. EMPA therapy enabled a reduction of G‐CSF dosage and contributed to improved overall clinical stability.

EMPA was initiated at a low dose (0.3 mg/kg/day) and gradually titrated up to 1.1 mg/kg/day based on clinical response, including reduction in infection frequency, improvement in oral lesions, and stabilization of neutrophil counts. Dose escalation was carefully guided by close monitoring for potential adverse effects, particularly hypoglycemia and urinary tract infections; no such complications were observed. Notably, 1.5‐anhydroglucitol (1.5‐AG) measurements were not available and treatment decisions were therefore based on clinical and hematological parameters.

Hepatic adenoma had been documented several years prior to EMPA initiation and showed progression during follow‐up, ultimately transforming into hepatocellular carcinoma (HCC), a recognized complication of GSD Ib and not related to EMPA therapy. The patient subsequently underwent liver transplantation at 18 years of age. EMPA was discontinued 2 days prior to surgery as a precaution against the risk of euglycemic ketoacidosis in the perioperative setting, particularly under conditions of fasting and surgical stress. Following an uncomplicated postoperative course, EMPA was reintroduced at the previously established maintenance dose approximately 3 weeks after transplantation, once the patient was clinically stable.

After transplantation, metabolic control improved significantly, allowing discontinuation of G‐CSF therapy and transition to an unrestricted diet. At last follow‐up, the patient remained clinically stable under EMPA, immunosuppressive therapy, and ongoing management of IBD (Table S2, **P7**).

A 29‐year‐old patient discontinued EMPA after 24 months of treatment due to pregnancy. Prior to EMPA initiation, she had received G‐CSF therapy at a dose of 3.4 μg/kg/day three times per week (10.2 μg/kg/week). During EMPA treatment, the frequency of G‐CSF was reduced to once per week and eventually discontinued. During pregnancy, EMPA was paused, and the patient resumed G‐CSF at the previous dose and frequency. She delivered a healthy baby; however, she developed sepsis in the postpartum period. After recovering, the patient attempted breastfeeding for 1 month, but due to a recurrence of severe infections, EMPA treatment was reinitiated, and breastfeeding was discontinued. Clinical follow‐up showed a subsequent improvement in infection frequency under EMPA therapy (Table S2, **P22**).

One patient with a highly complex clinical course died during the study period. She had been diagnosed with GSD Ib in early infancy due to hypoglycemia and a positive family history. Her disease course was marked by recurrent infections, gastrointestinal involvement, endocrine complications, and progressive renal disease secondary to amyloidosis. She developed end‐stage renal failure at 17 years of age and underwent kidney transplantation at 21 years.

Throughout childhood and adolescence, the patient experienced frequent infections, including recurrent otitis, lower respiratory tract infections, oral ulcers, and cellulitis. Despite some reduction in infection frequency in early adulthood, she continued to have a significant disease burden, compounded by IBD and immunosuppression following transplantation.

Empagliflozin was initiated at 24 years of age during hospitalization, starting at a low dose of 0.3 mg/kg/day due to the potential risk of hypoglycemia, particularly in the context of active gastrointestinal disease and possible malabsorption. On the first day of treatment, a mild decrease in blood glucose (67 mg/dL) was observed, which was attributed to reduced oral intake rather than a direct drug effect. Following stabilization of nutritional status, no further hypoglycemic episodes occurred. The dose was subsequently increased to 0.6 mg/kg/day within 3 days.

During the three‐month treatment period, the patient tolerated EMPA well, with no observed adverse effects such as hypoglycemia, lactic academia, or genitourinary infections. Importantly, no infectious episodes were recorded during this period, representing a notable clinical improvement compared to her previous course. As in other patients, 1.5‐AG measurements were not available, and treatment decisions were guided by clinical response and safety monitoring.

Despite the initial favorable response, the patient died suddenly at home approximately 3 months after treatment initiation. The exact cause of death could not be definitively established, as it occurred outside the hospital setting; however, it was considered most likely related to respiratory failure in the context of confirmed COVID‐19 infection and multiple underlying comorbidities, including advanced amyloidosis, immunosuppression, and chronic disease burden (Table S2, **P10**).

### 3.2 Treatment Details and Results

All data on EMPA and GCSF treatment are shown in Tables 1 and 2, Tables S1 and S2. EMPA treatment was temporarily paused in two patients (**P22**: pregnancy (9 months), breast feeding (1 month); **P7** peri‐liver transplantation period (2 days prior to surgery was stopped and 3 weeks after surgery was reintroduced)).

**TABLE 1:** Clinical and laboratory characteristics of the overall cohort, stratified by treatment groups.

		I: EMPA (*n* = 9)	II: G‐CSF (*n* = 7)	III: EMPA + GCSF (*n* = 16)	IV: Naïve (*n* = 10)	*p*
Age (years)	Median [IQR]	8.6 [6.5–14.0]	3.1 [2.7–5.1]	10.5 [6.5–16.2]	7.8 [5.0–12.4]	0.076
Sex	Male, *n* (%)	3 (33%)	6 (86%)	9 (56%)	6 (60%)	0.097
	Female, *n* (%)	6 (67%)	1 (14%)	7 (44%)	4 (40%)	
EMPA (mg/kg/day)	Initial/follow‐up dose Treatment duration (months)	0.34 ± 0.10/0.44 ± 0.14 27.6 ± 12.2	N/A	0.18 ± 0.09/0.45 ± 0.23 29.3 ± 9.4	N/A	N/A
G‐CSGF (μg/kg/day)	Dose Treatment duration (months)	N/A	0.54 ± 0.09 17.9 ± 0.9	1.40 ± 1.02 8.1 ± 5.6	N/A	N/A
ANC (1.5–8.0 × 10^9^/L)	Before treatment, median [IQR]	0.80 [0.6–1.2]	2.50 [1.5–3.5]	**1.00 [0.5–1.5]**	1.50 [1.0–2.0]	0.783
	During treatment, median [IQR]	1.80 [1.2–2.8]	2.20 [1.0–5.0]	**2.50 [1.5–4.5]**	2.00 [1.2–3.5]	
				***p* = 0.014**		
Hemoglobin (115–155 g/L)	Before treatment, median [IQR]	92 [84–103]	102 [94–111]	**103 [95–113]**	110 [100–121]	0.008
	During treatment, median [IQR]	105 [97–114]	98 [87–110]	**117 [107–128]**	107 [96–118]	
				***p* < 0.01**		
AST (< 35 U/L)	Before treatment, median [IQR]	40 [25–62]	58 [30–115]	42 [24–88]	55 [30–105]	0.13
	During treatment, median [IQR]	42 [28–65]	88 [38–240]	32 [22–46]	80 [58–108]	
ALT (< 45 U/L)	Before treatment, median [IQR]	38 [20–68]	55 [28–130]	35 [18–78]	48 [28–82]	0.42
	During treatment, median [IQR]	75 [52–112]	105 [48–195]	28 [16–46]	48 [30–75]	
ALP (40–129 U/L)	Before treatment, median [IQR]	132 [105–165]	208 [172–258]	138 [72–268]	168 [122–228]	0.77
	During treatment, median [IQR]	168 [140–208]	210 [188–235]	158 [118–215]	172 [128–230]	
Lactate (0.5–2.2 mmol/L)	Before treatment, median [IQR]	1.20 [0.7–2.0]	0.32 [0.2–0.5]	**0.55 [0.3–1.1]**	0.68 [0.4–1.2]	0.67
	During treatment, median [IQR]	1.10 [0.6–2.2]	0.52 [0.3–1.0]	**1.42 [0.7–2.8]**	0.90 [0.82–0.98]	
				***p* = 0.05**		
Triglyceride (< 1.7 mmol/L)	Before treatment, median [IQR]	4.20 [1.8–8.5]	3.40 [2.0–5.5]	5.20 [3.2–8.0]	4.80 [2.8–7.8]	0.81
	During treatment, median [IQR]	5.50 [3.2–8.8]	4.90 [3.2–7.2]	5.50 [3.5–8.5]	4.20 [2.0–8.5]	

*Note:* Significant results are presented in bold. Group sizes (*n*) are indicated in the column headers.

Abbreviations: ALP, alkaline phosphatase; ALT, alanine aminotransferase; AST, aspartate aminotransferase; Before treatment: before treatment with EMPA/G‐CSF or baseline for naïve patients; During treatment: during treatment with EMPA/G‐CSF or follow‐up period for naïve patients; EMPA: empagliflozin; F, female; G‐CSF, granulocyte‐colony stimulating factor; GGT, gamma glutamyl transferase; IQR, interquartile range; M, male; N/A: not applicable.

**TABLE 2:** Treatment and laboratory characteristics of pediatric patients, stratified by treatment subgroups.

		P‐I: EMPA (*n* = 7)	P‐II: GCSF (*n* = 5)	P‐III: GCSF+ EMPA (*n* = 11)	P‐IV: Naïve (*n* = 7)	*p*
Age (years)	Median [IQR]	6.8 [5.8–10.8]	4.7 [3.1–5.5]	10.0 [8.3–13.7]	8.2 [6.4–12.4]	0.161
Sex	Male, *n* (%)	2 (29%)	5 (100%)	6 (55%)	5 (71%)	0.169
	Female, *n* (%)	5 (71%)	0 (0%)	5 (45%)	2 (29%)	
Weight SDS	Before treatment	**−2.1 ± 0.9**	−1.5 ± 0.3	−2.8 ± 2.7	−1.7 ± 1.8	0.12
	During treatment	**−1.7 ± 0.9**	−1.9 ± 0.3	−1.4 ± 2.6	−1.7 ± 1.4	
		***p* = 0.05**				
Height SDS	Before treatment	−3.4 ± 1.8	−3.1 ± 1.5	−3.2 ± 3.2	−2.1 ± 1.3	0.49
	During treatment	−2.2 ± 3.2	−3.3 ± 0.3	−1.9 ± 3.4	−2.5 ± 2.2	
EMPA (mg/kg/day)	Initial/follow‐up dose Treatment duration (months)	0.32 ± 0.09/0.46 ± 0.16 27.5 ± 11.6	N/A	0.23 ± 0.13/0.54 ± 0.24 31.6 ± 12.3	N/A	N/A
G‐CSGF (μg/kg/day)	Dose Treatment duration (months)	N/A	0.43 ± 0.12 18.0 ± 0.0	1.60 ± 1.08 10.1 ± 7.0	N/A	N/A
Absolute neutrophil count (×10^9^/L; ref. 1.5–8.0)	Before treatment, median [IQR]	0.90 [0.7–1.3]	2.50 [1.5–4.0]	**0.90 [0.6–1.3]**	1.50 [1.0–2.0]	0.81
	During treatment, median [IQR]	2.50 [1.8–3.5]	3.80 [2.5–6.0]	**2.00 [1.5–3.0]**	2.00 [1.2–3.5]	
				***p* = 0.05**		
Hemoglobin (115–155 g/L)	Before treatment, median [IQR]	93 [82–106]	106 [102–110]	**106 [98–116]**	110 [100–121]	0.056
	During treatment, median [IQR]	108 [101–117]	102 [92–113]	**116 [105–128]**	107 [96–118]	
				***p* = 0.0120**		
Active inflammatory Bowel disease (6.99 ± 4.79)^a^	Before treatment (*n* patients)	**3**	**2**	*6*	**2**	**< 0.01**
	During treatment (*n* patients)	**0**	**0**	*0*	**5**	
Hospital admission (per patient)^b^	Before treatment mean ± SD	**5.8 ± 5.6**	1.3 ± 2.3	**5.3 ± 5.0**	0.5 ± 0.8	**0.024**
	During treatment, mean ± SD	**0.7 ± 1.2**	4.3 ± 4.9	**0.4 ± 1**	7 ± 11.3	
		***p* = 0.05**		***p* < 0.01**		
Infections (per patient)^c^	Before treatment, mean ± SD	**1.5 ± 1.7**	**0.3 ± 0.6**	**1.7 ± 1.4**	**1 ± 1.3**	**< 0.01**
	During treatment, mean ± SD	**0.5 ± 0.6**	**2.0 ± 1.0**	**0.3 ± 0.5**	**4 ± 3.0**	
		***p* < 0.01**	***p* = 0.05**	***p* < 0.01**	***p* = 0.049**	
AST (< 35 U/L)	Before treatment, median [IQR]	50 [35–75]	35 [18–62]	38 [18–72]	55 [28–108]	0.233
	During treatment, median [IQR]	55 [38–80]	82 [42–158]	32 [22–46]	80 [58–108]	
ALT (< 45 U/L)	Before treatment, median [IQR]	50 [28–85]	32 [15–62]	38 [22–62]	48 [28–82]	0.015
	During treatment, median [IQR]	78 [42–138]	98 [42–198]	27 [14–45]	48 [30–75]	
ALP (40–129 U/L)	Before treatment, median [IQR]	135 [108–172]	222 [178–278]	170 [138–212]	168 [122–228]	0.175
	During treatment, median [IQR]	185 [158–218]	205 [178–242]	182 [148–228]	172 [128–230]	
Lactate (0.5–2.2 mmol/L)	Before treatment, median [IQR]	1.52 [0.9–2.4]	1.52 [0.9–2.4]	0.58 [0.3–1.2]	0.68 [0.4–1.2]	0.848
	During treatment, median [IQR]	1.28 [0.6–2.5]	0.30 [0.18–0.52]	1.18 [0.5–2.5]	0.70 [0.3–1.2]	
Triglyceride: < 1.7 mmol/L (< 150 mg/dL)	Before treatment, median [IQR]	4.80 [2.2–9.5]	**3.10 [2.6–3.8]**	5.20 [2.5–9.5]	4.80 [2.8–7.8]	0.773
	During treatment, median [IQR]	6.50 [4.2–10.2]	**7.10 [5.5–9.0]**	5.80 [3.5–9.2]	8.80 [7.0–11.5]	
			***p* = 0.05**			

*Note:* Significant results are presented in bold. Group sizes (*n*) are indicated in the column headers. Italicized *p*‐values represent within‐group comparisons between pre‐treatment and during‐treatment values.

Abbreviations: ALP, alkaline phosphatase; ALT, alanine aminotransferase; AST, aspartate aminotransferase; Before treatment: before treatment with EMPA/G‐CSF or baseline for naïve patients; During treatment: during treatment with EMPA/G‐CSF or follow‐up period for naïve patients; EMPA: empagliflozin; F, female; G‐CSF, granulocyte‐colony stimulating factor; GGT, gamma glutamyl transferase; IQR, interquartile range; M, male; N/A: not applicable; SDS: standard deviation score.

^a^ Based on the weighted Pediatric Crohn's Disease Activity Index (wPCDAI), > 12.5 indicating active disease. Conversely, < 12.5 clinical remission.

^b^ Hospital admissions were recorded as the number of admissions per patient during the same observation period. For treated patients, the observation period corresponded to treatment duration, whereas for untreated patients it corresponded to the available follow‐up period.

^c^ Infection frequency was calculated as the number of clinically significant infections per patient during the defined observation period. Severe infections were defined as those requiring medical intervention and/or hospitalization.

**FIGURE 1:**
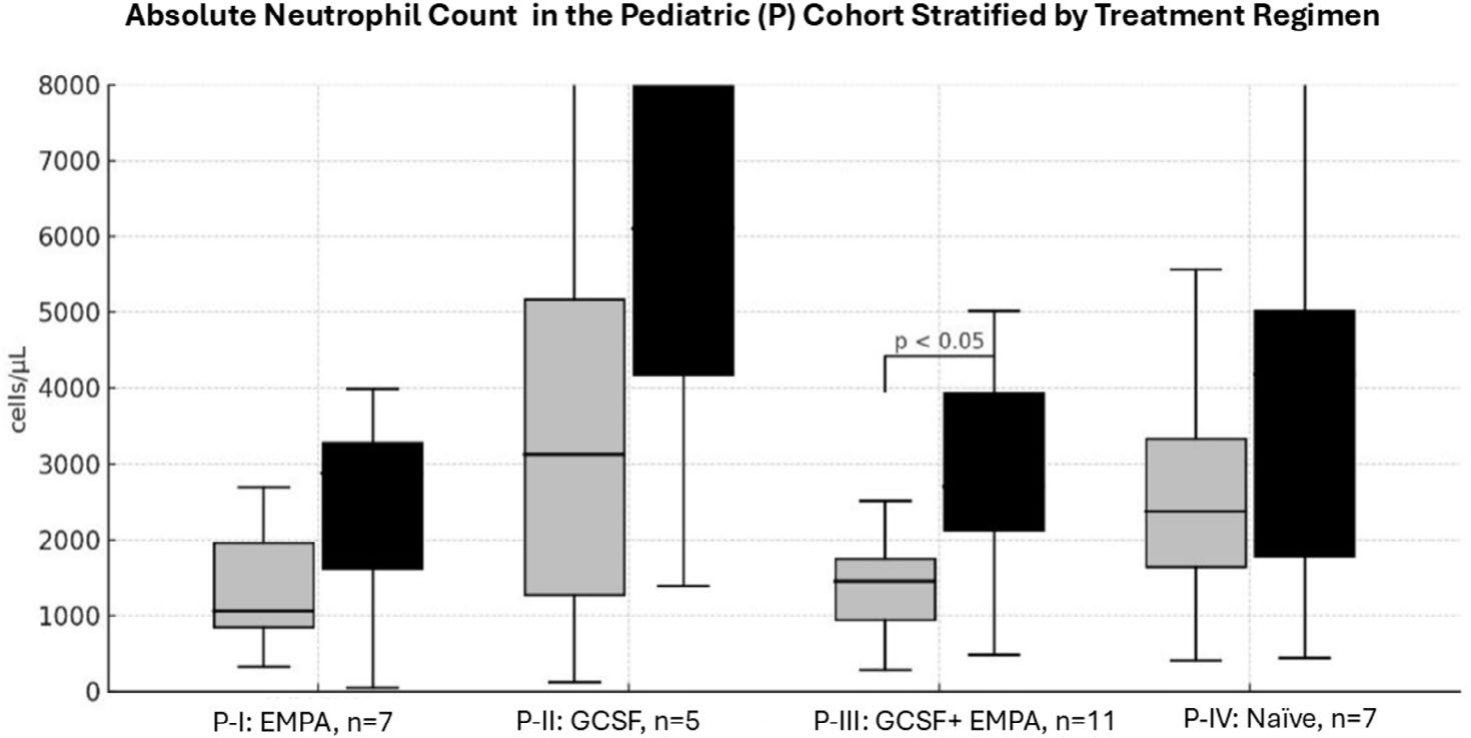
Absolute neutrophil count (ANC) in the pediatric (P) cohort stratified by treatment regimen. Pediatric patients were stratified into four groups: empagliflozin monotherapy (P‐I, *n* = 7), G‐CSF monotherapy (P‐II, *n* = 5), empagliflozin combined with G‐CSF (P‐III, *n* = 11), and treatment‐naïve patients (P‐IV, *n* = 7). Grey boxplots represent values before treatment, whereas black boxplots represent values during treatment.

**FIGURE 2:**
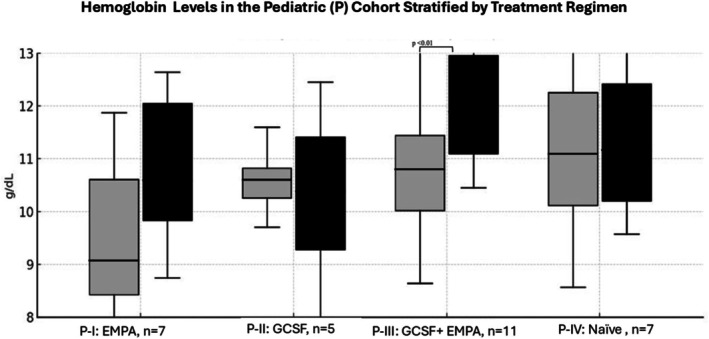
Hemoglobin (Hb) Levels in the pediatric (P) cohort stratified by treatment regimen. Data shown represent the pediatric subgroup (*n* = 30): P‐I (*n* = 7), P‐II (*n* = 5), P‐III (*n* = 11), and P‐IV (*n* = 7). Grey boxplots represent values before treatment, whereas black boxplots represent values during treatment.

A significant increase in ANC and hemoglobin was only seen in Group III, as well as in the subgroup P‐III (Figures 1 and 2) but not in the groups receiving EMPA or G‐CSF monotherapy.

In pediatric patients, a significant reduction in hospital admissions and infections was observed only in the groups receiving either EMPA alone or in combination with G‐CSF. Of note, G‐CSF treatment alone did lead to an increase in infections. In all patient subgroups with EMPA and/or G‐CSF treatment, IBD improved.

Hypoglycemia occurred in 22% of patients at baseline and 30% during follow‐up, with hospitalizations in three patients before and six patients during empagliflozin therapy. Glucosuria was observed in all patients in groups I and III.

In the evaluation of metabolic control, liver function tests showed no significant changes between four classes in the periods before and during the treatment (Table 1). Despite an observed increase in lactate in III (with *p* = 0.05).

In the pediatric cohort the increase in triglyceride levels in P‐II was statistically significant (*p* = 0.05) (Table 2).

During the follow‐up period, no statistically significant changes were observed in height‐SDS in P subgroup**s** (Table 2). However, a significant decrease in body weight‐SDS was detected in P‐I (*p* = 0.05).

In the evaluation of nutritional therapy P‐I demonstrated a significant increase in daily caloric intake (DCI) during EMPA treatment, rising from 1623.33 ± 500 kcal/day to 1809.33 ± 550 kcal/day (*p* = 0.027). Regarding macronutrient intake, I exhibited an increase in the percentage of DCI from carbohydrates, rising from 50.33% ± 7.87% of DCI before treatment to 59.42% ± 6.98% during treatment.

### 3.3 Adverse Events

The most severe adverse event was lactic acidosis, observed in two patients (P2 and P13, both in P‐III) and required hospitalization. In both cases, the episodes were associated with gastroenteritis and dehydration. EMPA treatment was paused for 3 to 5 days during these events.

Urinary tract infections (UTIs) were observed in two patients (7.4%) (P6 and P13, both in P‐III). Patient P13 required hospitalization due to persistent *Klebsiella pneumoniae* bacteriuria. The patient was subsequently placed on prophylactic treatment with trimethoprim‐sulfamethoxazole and fluconazole.

## 4 Discussion

This nationwide, multicenter study corroborates findings from previous studies showing that EMPA may serve as a safe and well‐tolerated therapy for individuals with GSD Ib. The use of EMPA was associated with a significant reduction in infections, hospital admissions, and IBD severity and overall nutritional status in pediatric patients.

One key finding of our study is the use of EMPA as a first‐line treatment in a multicenter cohort of nine patients. Also, in this subgroup the same effect of treatment was observed. Interestingly, there was no statistically significant increase in ANC. These findings suggest that EMPA may restore neutrophil function before or even in the absence of quantitative recovery, representing a clinically meaningful therapeutic benefit of first‐line EMPA treatment. For the first time, this study shows on a group level that most patients remain neutropenic on EMPA while having good neutrophil function and that normalization of ANC is only reached with the addition of G‐CSF. This can aid in clinical decision making.

Another important benefit of EMPA treatment documented in our study is that most patients (94%) were able to either discontinue or reduce their G‐CSF therapy after initiating EMPA, with 78% achieving full discontinuation. This finding aligns with growing evidence supporting the role of EMPA in improving neutrophil function and counts in patients with GSD Ib [6, 7, 9, 10, 11, 12, 13, 14, 17, 18, 19, 20, 21, 22, 23, 24, 25, 26, 27]. Discontinuation of G‐CSF should be considered in all individuals, particularly considering the potential long‐term risks associated with its use, including myelodysplastic syndrome and leukemia [23].

In this patient cohort, we also encountered and managed three special clinical situations that may arise in individuals with GSD Ib: liver transplantation (LTx), kidney transplantation, and pregnancy. In line with current general recommendations, individualized treatment decisions were made [24, 28, 29]. EMPA was prescribed by metabolic specialists, with treatment initiation individualized according to age and clinical status; it was typically hospital‐based in younger patients and outpatient in stable older individuals, with continuous glucose monitoring recommended where available. EMPA was paused peri‐operatively in the patient undergoing LTx and subsequently restarted after recovery, whereas it was temporarily withheld during pregnancy and lactation in another patient. The decision regarding temporary interruption around LTx was made while acknowledging the ongoing discussion in the literature, particularly the report by Murko et al. which argues for the discontinuation of SGLT2 inhibitors following liver transplantation in GSD Ib [29]. This aligns with our peri‐operative approach, while the consensus paper [24] highlights that continuation of SGLT2 inhibitors may be feasible after transplantation (section 5.1.2). In our liver transplantation case, empagliflozin had contributed to sustained improvement in neutropenia and infection‐related complications before transplantation, allowing reduction of G‐CSF therapy. Perioperative discontinuation was primarily guided by the known risk of euglycemic ketoacidosis in the context of fasting, surgical stress, and general anesthesia, and treatment was safely reintroduced after clinical stabilization.

For the pregnant patient, EMPA was discontinued due to the lack of long‐term safety data in this setting. This contrasts with the encouraging case reports by Grunert et al. [30], which reported two successful pregnancies and the first use of EMPA during pregnancy in GSD Ib, with positive outcomes [30]. Initial efforts were made to continue treatment with G‐CSF alone during both pregnancy and lactation. However, due to severe infections and the patient's own preference, G‐CSF was eventually discontinued and EMPA was reintroduced. Consequently, breastfeeding was ceased, and the infant was transitioned to formula feeding. Particular attention was paid to the patient who had undergone kidney transplantation, with the patient continuing therapy under close monitoring. Similarly, in the kidney transplantation case, empagliflozin was well tolerated despite multiple comorbidities, including amyloidosis, IBD, and immunosuppression. The transient hypoglycemia observed early after treatment initiation was considered more likely related to reduced oral intake during active gastrointestinal disease than to a direct drug effect. This case underlines the importance of careful patient selection, close monitoring, and individualized dose titration, particularly in patients with complex multisystem involvement. In the absence of routine 1.5‐anhydroglucitol measurements in our cohort, treatment decisions in such complex cases were mainly guided by clinical response, neutrophil counts, and safety parameters. Collectively, these observations provide practical real‐world insights for clinicians managing GSD Ib patients in the settings of transplantation, pregnancy, and other complex clinical scenarios.

This finding suggests that EMPA may have a more pronounced effect on controlling infections and IBD symptoms compared to G‐CSF alone. This potential superiority of EMPA in managing GSD‐related IBD was also recently documented and highlighted by Li et al. [14].

In our cohort, a significant improvement in hemoglobin levels was observed in I and P‐III. This increase, which was already evident in interim analyses and persisted through the end of the study, suggests a sustained benefit and is consistent with findings reported in the literature [7, 10, 11, 12, 13, 14]. Anemia is common in patients with GSD Ib, potentially due to the accumulation of 1,5‐anhydroglucitol‐6‐phosphate (1,5‐AG6P) and, in the case of anemia, also due to iron malabsorption associated with IBD. SGLT2 inhibitors, such as EMPA, enhance urinary excretion of 1,5‐AG, which has been linked to the resolution of anemia in many cases [5, 6].

Hypoglycemia (as a manifestation of the primary disease) was observed in 22% of patients prior to the initiation of EMPA treatment and increased to 30% during the treatment period, either as a clinical feature of the disease or a potential adverse event associated with the drug. In light of these findings—and given that the literature reports an average hypoglycemia rate of approximately 18% with EMPA [9]—careful monitoring for hypoglycemia during EMPA therapy is strongly recommended [10, 31, 32].

In terms of nutritional status, the significant increase in body weight‐SDS observed in the first line EMPA‐treated group may reflect improved nutritional status, likely due to the resolution of IBD‐related symptoms and better gastrointestinal function reported by Li et al. [14] and Kose et al. [12]. Before treatment, many GSD Ib patients had difficulty tolerating starch therapies due to IBD [23].

In our patient series, the incidence of UTI was reported as 7.4%, which is consistent with the 7% reported by Grunert et al. [9] in a cohort of 112 patients. Importantly, this rate is also comparable to UTI incidences reported in large diabetes cohorts treated with SGLT2 inhibitors, where rates typically range between 5% and 9% and are not consistently higher than in control groups [28]. However, this is markedly different from the 60% incidence reported by Köse et al. [12] in a much smaller cohort of 10 patients. The notably higher rate in that series may be attributed to the use of EMPA in two daily doses, with the second dose potentially causing nocturnal glucosuria, which could act as a predisposing factor for UTIs. Therefore, the recent recommendation by Grunert et al. [23] to use a single daily dose of EMPA may be clinically significant and should be taken into consideration.

### 4.1 Limitations

While this study provides novel data, particularly being the first to evaluate patients treated with EMPA as a first‐line therapy in comparison with those not receiving the drug, several limitations should be acknowledged. Foremost, this was an observational, retrospective study, not a randomized controlled trial. Consequently, the decision to initiate therapy with EMPA, G‐CSF, or both was at the discretion of the treating physician and not determined by a standardized protocol. In addition, several constraints regarding available patient data limited our analysis. We were unable to assess the effect of EMPA on 1,5‐anhydroglucitol (1,5‐AG) levels, as this analysis is not routinely available in clinical practice. Furthermore, reliable longitudinal data on fecal calprotectin—another potentially valuable non‐invasive biomarker for intestinal inflammation—were inconsistently available across the cohort.

## 5 Conclusions

This national, multicenter study provides real‐world evidence supporting the use of EMPA as a safe and well‐tolerated first‐line therapy for patients with glycogen storage disease type Ib (GSD Ib). EMPA monotherapy was associated with significant and sustained improvements in key clinical outcomes, including reductions in hospital admissions, infection frequency, and IBD severity, as well as enhancements in overall nutritional status. Based on clinical experience from this cohort, once‐daily EMPA as a first‐line therapy may offer both practical and therapeutic advantages.

## Author Contributions

S.K.U. guarantor for the article. S.K.U., S.B.W., and D.Y.K. conceptualized and designed the study, coordinated and supervised data collection, drafted the initial manuscript, and reviewed and revised the manuscript. A.E. performed the statistical analysis. All authors carried out the clinical analyses, collected diagnoses, and treatment data, as well as reviewed and revised the manuscript. All authors approved the final manuscript as submitted and agree to be accountable for all aspects of the work.

## Funding

The authors have nothing to report.

## Ethics Statement

The human studies described here were conducted with the approval of the Ege University Medical Ethics Committee (E‐99166796‐050.04‐1 717 668). Open access funding provided by the Scientific and Technological Research Council of Türkiye (TÜBİTAK).

## Consent

All volunteers who participated in this study provided appropriate informed consent under Ege University Medical Ethics Committee protocol (E‐99166796‐050.04‐1 717 668) and adhered to the tenets of the Declaration of Helsinki.

## Conflicts of Interest

The authors declare no conflicts of interest.

## Supporting information


**Table S1:** Age distribution of the patients across subgroups.

**Table S2:** Overview of demographic data, molecular findings, and treatment characteristic.

**Figure S1:** The distribution of study sites based on the geographical regions of the cities, with each circle representing a study site.

## Data Availability

Data supporting the results are available in the manuscript and Supporting Information. Additional information is available upon request.
