# Supplementary material for: Shifting Towards Empagliflozin First‐Line Therapy in Glycogen Storage Disease Type Ib: A Nationwide Real‐World Study
Source: J Inherit Metab Dis. 2026 May 3;49:e70198. doi: 10.1002/jimd.70198 (PMC13136049; doi:10.1002/jimd.70198)
Supplement: Supplementary file 1 — Table S1: Age distribution of the patients across subgroups. [file JIMD-49-0-s002.docx]

**Supplementary Table 1**: Age distribution of the patients across subgroups

|  | **I: EMPA** | **II: G-CSF** | **III: EMPA + G-CSF** | **IV: Naive** | **Total Numbers** |
| --- | --- | --- | --- | --- | --- |
| **Infants** | 0 | 2 | 2 | 2 | 6 |
| **Pediatric** | 7 | 5 | 11 | 7 | 30 |
| **Adults** | 2 | 0 | 3 | 1 | 6 |
| **Total numbers** | 9 | 7 | 16 | 10 | 42 |

**Abbreviations:** EMPA: Empagliflozin; G-CSF, Granulocyte-Colony Stimulating Factor, Infants: 0-36 months, Pediatric patients: 37 months -18 years; Adults: > 18 years
