# Supplementary material for: Shifting Towards Empagliflozin First‐Line Therapy in Glycogen Storage Disease Type Ib: A Nationwide Real‐World Study
Source: J Inherit Metab Dis. 2026 May 3;49:e70198. doi: 10.1002/jimd.70198 (PMC13136049; doi:10.1002/jimd.70198)
Supplement: Supplementary file 2 — Table S2: Overview of demographic data, molecular findings, and treatment characteristic. [file JIMD-49-0-s001.docx]

**Supplementary Table 2:** Overview of Demographic Data, Molecular Findings, and Treatment Characteristic

| **Patient Number** | **Age**  **(at beginning)** | **Sex** | ***SLC37A4* (NM_001164277.2)**  **variant 1** | ***SLC37A4* (NM_001164277.2)**  **variant 2** | **EMPA Treatment** | | | **G-CSF Treatment** | | **Treatment**  **Subgroups** | |
| --- | --- | --- | --- | --- | --- | --- | --- | --- | --- | --- | --- |
|  |  |  |  |  | **Initial/**  **Follow-up Dose**  (mg/kg/ day) | **Titration Period**  (Weeks)  **Doses/Day** | **Treatment**  **Duration**  (months) | **Dose**  (μg/kg/day)  dosages/regimens | **Discontinuation Time (D)** (months)**/**  **Continuing (C)** | **Medicine**  **Sub**  **group** | **Age**  **Sub**  **group** |
| P1 | 25y | M | c.773C>G  (p.Ser258*) | c.773C>G  (p.Ser258*) | 0.14/  0.28 | 2  1 | 34 | N/A | N/A | I | A-I |
| P2 | 6y 6mo | F | c.625G>C  (p.Lys208Asn) | c.625G>C  (p.Lys208Asn) | 0.25/  0.25 | 1  1 | 38 | N/A | N/A | I | P-I |
| P3 | 10y | M | c.381+1G>C | c.381+1G>C | 0.16/  0.33 | 2  1 | 41 | 2.5  3×/week | S  2 mo | III | P-III |
| P4 | 4y | F | c.406+1G>A | c.406+1G>A | 0.31/  0.31 | 1  1 | 43 | N/A | N/A | I | P-I |
| P5 | 14y | M | c.1042_1043del  (p.Leu348Valfs*53) | c.1042_1043del  (p.Leu348Valfs*53) | 0.19/  0.29 | 2  1 | 15 | N/A | N/A | I | P-I |
| P6 | 16y | F | c.365G>A  p.Gly122Glu | c.365G>A  p.Gly122Glu | 0.08/  0.35 | 4  1 | 47 | 0.91  3×/week | D  2 mo | III | P-III |
| P7* | 11y 5mo | M | c.365G>A  (p.Gly122Glu) | c.365G>A  (p.Gly122Glu) | 0.25/  1 | 4  1 | 24 | 4  3×/week | C  18 mo | III | P-III |
| P8 | 11y | F | c.365G>A  (p.Gly122Glu) | c.365G>A  (p.Gly122Glu) | 0.26/  0.6 | 2  1 | 45 | 1.8  2×/week | D  18 mo | III | P-III |
| P9 | 9 y 7 mo | M | c.1042_1043del  (p.Leu348Valfs*53) | c.1042_1043del  (p.Leu348Valfs*53) | 0.5/  1 | 2  1 | 30 | 0.37  3×/week | D  18 mo | III | P-III |
| P10 ** | 24y | F | c.1042_1043del  p.Leu348Valfs*53 | c.1042_1043del  p.Leu348Valfs*53 | 0.3/  0.6 | 2  1 | 3 | No | No | I | A-I |
| P11 | 14 mo | M | c.904+3_904+6del | c.904+3_904+6del | 0.4  0.5 | 2  1 | 25 | 2.1  3×/week | D  18 mo | III | Inf-III |
| P12 | 5y | F | c.1042_1043del  (p.Leu348Valfs*53) | c.1042_1043del  (p.Leu348Valfs*53) | 0.3  0.3 | 1  1 | 25 | 3.2  3×/week | D  18 mo | III | P-III |
| P13 | 5y | M | c.24T>G;c.981delC (cis)  p.Tyr8*;p.Lys328Serfs*25(cis) | c.24T>G;c.981delC (cis)  p.Tyr8*;p.Lys328Serfs*25(cis) | 0.52  0.52 | 1  1 | 36 | No | No | I | P-I |
| P14 | 20y | M | c.365G>A  (p.Gly122Glu) | c.365G>A  (p.Gly122Glu) | 0.08  0.43 | 4  2 | 14 | 0.22  3×/week | D  12 mo | III | A-III |
| P15 | 17y | F | c.365G>A  (p.Gly122Glu) | c.365G>A  (p.Gly122Glu) | 0.1  0.2 | 2  1 | 24 | 0.12  3×/week | D  2 w | III | P-III |
| P16 | 13y | F | c.365G>A  (p.Gly122Glu) | c.365G>A  (p.Gly122Glu) | 0,13  0.38 | 2  1 | 24 | No | No | I | P-I |
| P17 | 16y | M | c.925delG  (p.Ala309Leufs*3) | c.925delG  (p.Ala309Leufs*3) | 0,12  0.37 | 2  1 | 36 | 0.33  3×/week | D  2 mo | III | P-III |
| P18 | 29y | F | c.81T>A  (p.Asn27Lys) | c.81T>A  (p.Asn27Lys) | 0.1  0.4 | 2  2 | 15 | 0.4  3×/week | D  2 mo | III | A-III |
| P19 | 9y 10mo | M | c.1042_1043del  (p.Leu348Valfs*53) | c.1042_1043del  (p.Leu348Valfs*53) | 0.3  0.89 | 4  2 | 30 | 1.5  intermittent | D  2 w | III | P-III |
| P20 | 7y | F | c.288G>A  (p.Trp96*) | c.288G>A  (p.Trp96*) | 0.22  0.45 | 4  2 | 18 | 1.9  3×/week | C  1×/week | III | P-III |
| P21 | 4y 5mo | M | c.82C>A  (p.Arg28Ser) | c.82C>A  (p.Arg28Ser) | 0.22  0.31 | 4  1 | 36 | 3  3x /week | C  1x /week | III | P-III |
| P22*** | 29y | F | c.742C>T  (p.Gln248*) | c.742C>T  (p.Gln248*) | 0.1  0.4 | 4  1 | 24 + 4 | 3.4  3x /week | C  2 mo | III | A-III |
| P23 | 2y 7mo | M | c.83G>A  (p.Arg28His) | c.83G>A  (p.Arg28His) | 0.1  0.5 | 4  1 | 9 | 5  3x /week | D  3 mo | III | Inf-III |
| P24 | 6y 10mo | F | c.365G>A  (p.Gly122Glu) | c.365G>A  (p.Gly122Glu) | 0.3  0.5 | 2  1 | 12 | N/A | N/A | I | P-I |
| P25 | 8y 7mo | F | c.365G>A  (p.Gly122Glu) | c.365G>A  (p.Gly122Glu) | 0.4  0.7 | 2  1 | 12 | N/A | N/A | I | P-I |
| P26 | 12y 5mo | M | c.898C>T (p.Arg300Cys) | c.898C>T (p.Arg300Cys) | N/A | N/A | N/A | N/A | N/A | IV | P-IV |
| P27 | 3y 1mo | M | c.1042_1043del CT(p.Gly149Glnfs*64) | c.444delTinsCCA (p.Gly149Glnfs*64) | N/A | N/A | N/A | 0.50  3×/week | C  3×/week | II | P-II |
| P28 | 5y 6mo | M | c.1016G>A (p.Gly339Asp) | c.1016G>A (p.Gly339Asp) | N/A | N/A | N/A | 0.30  3×/week | C  3×/week | II | P-II |
| P29 | 5y 7mo | M | c.1042_1043del (p.Leu348Valfs*53) | c.444delTinsCCA(p.Gly149Glnfs*64) | N/A | N/A | N/A | N/A | N/A | IV | P-IV |
| P30 | 3y 1mo | M | c.1042_1043del (p.Leu348Valfs*53) | c.444delTinsCCA (p.Gly149Glnfs*64) | N/A | N/A | N/A | 0.40  3×/week | C  3×/week | II | P-II |
| P31 | 4y 10mo | M | c.1042_1043del (p.Leu348Valfs*53) | c.1042_1043del (p.Leu348Valfs*53) | N/A | N/A | N/A | N/A | N/A | IV | P-IV |
| P32 | 13y 1mo | M | c.1243C>T ( p.Arg415*) | c.1243C>T (p.Arg415*) | N/A | N/A | N/A | N/A | N/A | IV | P-IV |
| P33 | 4y 8mo | M | c.1016G>A (p.Gly339Asp) | c.1016G>A (p.Gly339Asp) | N/A | N/A | N/A | 0.50  3×/week | C  3×/week | II | P-II |
| P34 | 12y 8mo | M | c.382T>C (p.Trp128Arg) | c.382T>C (p.Trp128Arg) | N/A | N/A | N/A | 0.50  3×/week | C  3×/week | II | P-II |
| P35 | 8y 3mo | F | c.364G>A (p.Gly122Arg) | c.364G>A (p.Gly122Arg) | N/A | N/A | N/A | N/A | N/A | IV | P-IV |
| P36 | 22 y | F | c.1042_1043del (p.Leu348Valfs*53 | c.1042_1043del (p.Leu348Valfs*53) | N/A | N/A | N/A | N/A | N/A | IV | A-IV |
| P37 | 2y 3mo | F | c.288G >A (p.Trp96*) | c.288G >A (p.Trp96*) | N/A | N/A | N/A | 0.30  2×/week | 0.30  2×/week | II | Inf-II |
| P38 | 7y 3mo | M | c.699C>G (p.Tyr233*) | c.699C>G (p.Tyr233*) | N/A | N/A | N/A | N/A | N/A | IV | P-IV |
| P39 | 5mo | F | c.1042_1043del (p.Leu348Valfs*53) | c.1042_1043del (p.Leu348Valfs*53) | N/A | N/A | N/A | N/A | N/A | IV | Inf-IV |
| P40 | 10mo | M | c.1042_1043del (p.Leu348Valfs*53) | c.1042_1043del (p.Leu348Valfs*53) | N/A | N/A | N/A | N/A | N/A | IV | Inf-IV |
| P41 | 12y 4mo | F | c.1042_1043del (p.Leu348Valfs*53) | c.1042_1043del (p.Leu348Valfs*53) | N/A | N/A | N/A | N/A | N/A | IV | P-IV |
| P42 | 2y | M | c.699C>G (p.Tyr233*) | c.699C>G (p.Tyr233*) | N/A | N/A | N/A | 1  3×/week | C  3×/week | II | Inf-II |

Abbreviations: y, years; mo, months; w, week; M, male; F, female; EMPA, Empagliflozin**;**  G-CSF, Granulocyte-Colony Stimulating Factor; Discontinuation, D; C, Continuing; N/A, Not applicable; I: EMPA as first-line monotherapy, II: G-CSF monotherapy, III: G-CSF plus EMPA, and IV: neither G-CSF nor EMPA (treatment-naïve), P, Pediatric (36 mo-18yrs); A, Adult (>18 yrs); Inf, Infant (3mo-36mo)

*P7: Two years on EMPA with good neutrophil response. A long-standing hepatic adenoma progressed to HCC (a known GSD Ib complication). EMPA was paused for ≈ 3 weeks during the peri-transplant period and restarted after recovery.

**P10: History of renal failure and kidney transplantation. Frequent infections in earlier years. EMPA started at age 24 with no adverse effects and no infections during treatment. In the third month, he developed acute illness at home due to suspected viral infection (COVID-19), was admitted to intensive care, and died from cardiac arrest secondary to respiratory failure.

***P22: EMPA stopped during pregnancy after 24 months of therapy; G-CSF resumed at prior dose. She delivered a healthy baby, developed postpartum sepsis, and after one month of breastfeeding experienced recurrent infections. EMPA was restarted and breastfeeding discontinued.
