# Supplementary figures and images for: Shifting Towards Empagliflozin First‐Line Therapy in Glycogen Storage Disease Type Ib: A Nationwide Real‐World Study
Source: J Inherit Metab Dis. 2026 May 3;49:e70198. doi: 10.1002/jimd.70198 (PMC13136049; doi:10.1002/jimd.70198)

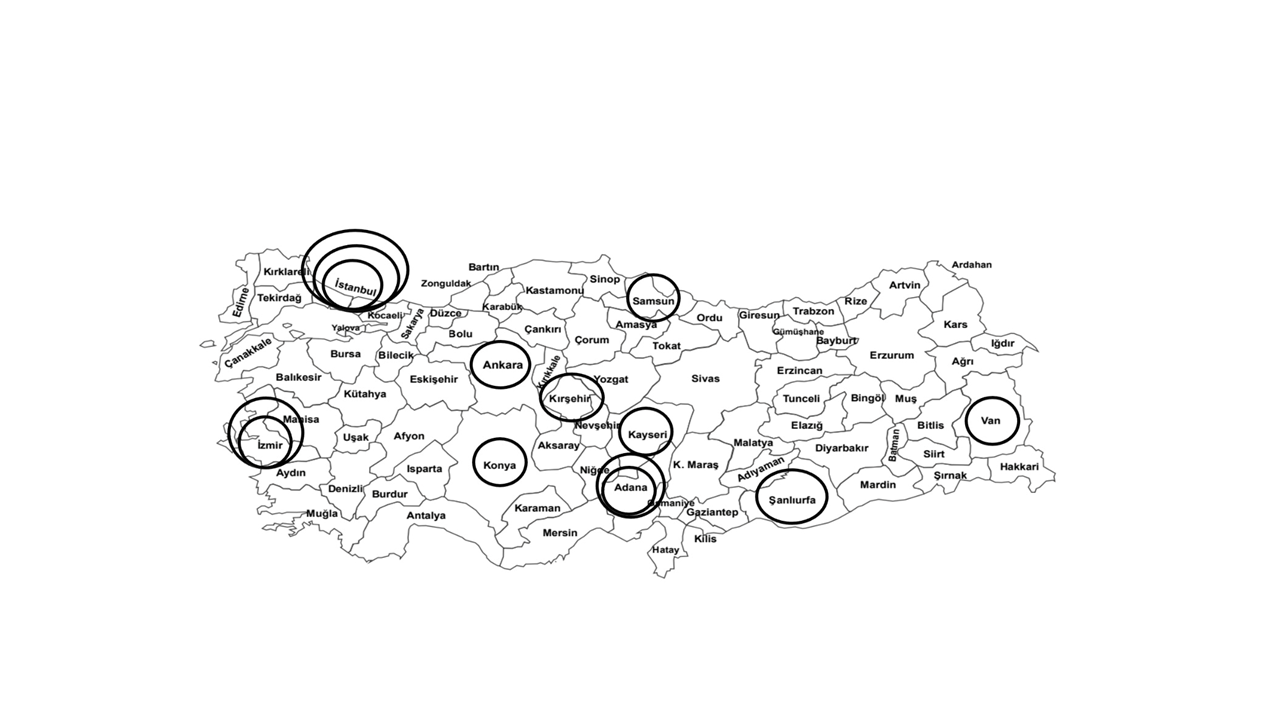

Supplement: Supplementary file 3 — Figure S1: The distribution of study sites based on the geographical regions of the cities, with each circle representing a study site. [file JIMD-49-0-s003.tif]
